# Age-specific vulnerabilities in paediatric dental emergencies before, during, and after COVID-19 lockdown: a retrospective comparative analysis with emphasis on early childhood (0–3 years)

**DOI:** 10.1007/s40368-025-01088-5

**Published:** 2025-07-29

**Authors:** D. Atia Joachim, D. Haim, E. Shapiro, T. Asbi, M. V. Joachim

**Affiliations:** 1 Ariel University, Ariel, Israel; 2 Maccabi Health Care Services, Tel Aviv, Israel; 3 Guarulhos University, Guarulhos, Brazil; 4 Tel Aviv University, Tel Aviv, Israel

**Keywords:** COVID-19, Pediatric dentistry, Dental emergency, Child, Preschool, Health services accessibility, Primary teeth

## Abstract

**Purpose:**

To examine the unique vulnerability patterns of very young children (0–3 years) in dental emergencies before, during, and after the COVID-19 lockdown period, compared to older paediatric patients. The study aimed to identify age-specific risk factors and treatment needs that demonstrate increased susceptibility to severe dental emergencies during healthcare crises.

**Methods:**

A retrospective analysis of 6,024 emergency dental visits of children under 12 years was conducted, comparing three periods: pre-COVID (March–May 2019), during the first lockdown (March–May 2020), and post-lockdown (March–May 2021). Data from a major Israeli healthcare provider's dental clinics were analysed for age-specific patterns in emergency presentations, treatment requirements, and clinical outcomes.

**Results:**

Children aged 0–3 years showed distinct emergency patterns during lockdown, with significantly higher rates of pulpal pain (51.2% vs. 42.1% pre-COVID) and dental abscess (24.8% vs. 19.5% pre-COVID). This age group experienced the highest proportion of invasive treatments (24.8% requiring extraction) compared to other age groups. The mean age of emergency presentations decreased significantly during lockdown (6.2 years vs. 7.1 years pre-COVID, *p* < 0.001), with the 0–3 age group showing the most marked increase in severity of presentations.

**Conclusion:**

Very young children (0–3 years) demonstrated specific vulnerabilities during the healthcare crisis, characterized by increased susceptibility to severe dental emergencies, higher rates of invasive interventions (44.5% vs. 30.6% pre-COVID), and disproportionate representation in emergency presentations. These findings demonstrate age-specific vulnerabilities that require targeted emergency protocols and preventive strategies during future healthcare crises.

**Supplementary Information:**

The online version contains supplementary material available at 10.1007/s40368-025-01088-5.

## Introduction

The COVID-19 pandemic created unprecedented challenges for healthcare systems worldwide, with significant implications for dental services and access to oral healthcare. Following the identification of the first case in Israel on February 21, 2020, the Israeli government implemented stringent measures to contain the virus spread, including a nationwide lockdown beginning March 16, 2020 (Abramovitz et al. 2020). During this period, dental clinics were restricted to providing emergency care only, creating a unique natural experiment to assess the minimum threshold of essential dental services required for different population groups (Meng et al. 2020).

Young children represent a particularly vulnerable population in the context of dental emergencies. The anatomical structure of primary teeth, characterized by thinner enamel and wider root canals, allows more rapid progression of dental caries and pulpal inflammation compared to permanent dentition (Luzzi et al. 2021). Additionally, very young children (ages 0–3) have limited ability to communicate pain severity, often leading to delayed diagnosis and treatment (Samuel et al. 2019). These factors, combined with an immature immune system, can result in faster development of infections and more severe complications in this age group (Sudri et al. 2024).

During normal circumstances, dental emergencies in children commonly stem from pulpal origin pain, trauma, and soft tissue injuries (Fux-Noy et al. 2021). However, several studies have suggested that the pattern and severity of paediatric dental emergencies changed significantly during the COVID-19 pandemic. Research from India found that nearly 80% of paediatric emergency dental visits during lockdown were related to pulpal pain or pathology (Kumar et al. 2021), while a study from Poland reported a three- to four-fold increase in abscess drainages and temporary restorations among children (Olszewska et al. 2021).

The existing literature reveals some concerning trends regarding emergency dental care for children during the pandemic. Studies from Switzerland, Saudi Arabia, and Argentina have reported a notable decrease in the average age of patients seeking emergency dental care during lockdown periods (Alzahrani et al. 2021; Eggmann et al. 2021; Rodriguez et al. 2022). However, while the impact of the pandemic on dental services generally has been extensively studied, research specifically examining age-specific vulnerabilities among different paediatric population segments remains limited.

Previous research has established that children's oral healthcare needs differ substantially based on developmental stage, with varying susceptibility to dental diseases and capacity to cooperate with treatment (Goswami et al. 2021). Studies from the United Arab Emirates and Jordan suggest that delay in dental treatment during lockdown periods disproportionately affected young children, with increased severity of conditions requiring more invasive interventions (Obeidat et al. 2020; Remmani et al. 2023). However, few studies have specifically focused on quantifying the unique emergency dental needs and treatment patterns of very young children (0–3 years) during healthcare crises. While studies on general paediatric dental emergencies during COVID-19 have been conducted in Israel (Elalouf et al. 2022; Fux-Noy et al. 2021), research specifically examining the unique vulnerability patterns of different age groups, particularly the youngest patients (0–3 years), has been lacking in the Israeli context. This represents a significant gap in our understanding of age-specific emergency dental needs in the Israeli healthcare system, particularly during crisis situations.

Understanding the specific vulnerability patterns of different age groups, particularly the youngest patients, is crucial for effective emergency dental service planning during future healthcare disruptions. The lockdown period offers a rare opportunity to identify the minimum essential services required for this vulnerable population by analysing genuine emergency needs isolated from routine dental visits (Dave et al. 2020).

The aim of this study was to examine the unique patterns and vulnerabilities of very young children (0–3 years) in dental emergencies during the COVID-19 lockdown period compared to older paediatric patients. By analysing the age-specific distribution of emergency presentations, treatment needs, and clinical outcomes, this research seeks to identify factors contributing to increased vulnerability in this young age group and to inform age-appropriate emergency dental protocols for future healthcare crises. Additionally, insights gained from analysing these vulnerability patterns during an extreme situation can enhance our understanding of age-specific dental needs in routine clinical settings, potentially improving preventive strategies and treatment approaches for very young children in everyday practice.

## Materials and methods

### Study design and ethical approval

This retrospective observational study was conducted and reported according to the Strengthening the Reporting of Observational Studies in Epidemiology (STROBE) guidelines for cross-sectional studies. It analysed data collected from the computerized dental care information system of Maccabi Dent, a major dental care provider for Maccabi Healthcare Services, which serves approximately 25% of Israel's population. The study was approved by the Institutional Helsinki Committee of Maccabi Healthcare Services and the Ethics Committee of Ariel University. Data were extracted systematically from the computerized dental care information system using predefined emergency visit classification codes and age parameters. All data were fully anonymized prior to analysis, with all identifying patient information removed while preserving relevant research variables such as age, sex, geographic area, clinical diagnoses, and treatment modalities.

### Study periods and population

The study examined three parallel time periods: pre-COVID period (March 25, 2019–May 4, 2019), first lockdown period (March 25, 2020–May 4, 2020), and post-lockdown/living alongside COVID period (March 25, 2021–May 4, 2021). These periods were selected to allow direct comparison between the unique circumstances of the first COVID-19 lockdown and equivalent time frames before and after, controlling for seasonal variations in dental emergencies.

The study population included all children under 12 years of age who sought emergency dental treatment at Maccabi Dent clinics during the three study periods. The 12-year age limit was chosen because beyond this age, most patients present with permanent dentition only, with minimal differences in dental characteristics compared to adults. For detailed age-specific analysis, patients were categorized into four age groups: 0–3 years, 4–6 years, 7–9 years, and 10–12 years.

### Participant selection and eligibility criteria

Inclusion Criteria:

- Children under 12 years of age at the time of treatment
- Emergency dental visits to Maccabi Dent clinics during the three specified study periods
- Complete documentation in the computerized dental care information system

Exclusion Criteria:

- Children 12 years of age or older
- Routine dental appointments or planned treatments
- Incomplete medical records lacking essential data (age, sex, reason for visit, or treatment provided)

### Emergency care delivery system during lockdown

During normal operations, Maccabi Dent operated 53 dental clinics nationwide. During the first lockdown, only 29 branches with 84 dentists remained operational to provide emergency care for both adults and children throughout Israel. Selection of operational clinics was based on geographic accessibility to ensure adequate coverage for the population.

During the lockdown period, Maccabi Dent operated a dedicated telephone triage centre where dental professionals evaluated patients' chief complaints, reviewed their dental records, and coordinated appointments at the nearest available clinics. Emergency conditions were defined in accordance with international guidelines (Royal College of Physicians and Surgeons of Glasgow 2020), with an emergency requiring treatment within 60 min and an urgent case requiring treatment within 24 h in the absence of worsening.

### Study variables

For the purpose of this study, we define vulnerability as the increased susceptibility of specific age groups to severe dental emergencies and adverse outcomes due to biological, developmental, and clinical factors that are intrinsic to their age group. This operational definition guided our analysis of age-specific patterns in emergency presentations, treatment requirements, and clinical outcomes.

The study analysed demographic, clinical, and treatment-related variables. Demographic data included patient age (categorized into four groups: 0–3, 4–6, 7–9, and 10–12 years) and sex. Age 12 was selected as the upper limit because beyond this age, most patients present with permanent dentition only, with minimal differences in dental characteristics compared to adults.

The main clinical variables analysed were reasons for emergency visits and types of treatment provided. Reasons for emergency visits were categorized into five mutually exclusive categories based on established clinical classifications in paediatric dental emergency literature:

1. Pain of dental origin (related to deep caries, pulpitis, or tooth sensitivity)
2. Pain of soft tissue origin (including gingival pain, ulcers, and oral wounds)
3. Repair of existing treatment (issues with previous dental work)
4. Dental trauma (including fractures, luxations, and avulsions)
5. Swelling and abscess in the oral cavity (infection-related presentations)

This categorization was performed by the primary researcher (DAJ), a paediatric dentistry specialist, based on the documented chief complaint and clinical diagnosis in the electronic dental records. These categories were selected to represent the full spectrum of common emergency presentations encountered in paediatric dental practice while ensuring mutually exclusive classifications.

Treatment variables were classified into six main categories based on clinical invasiveness and resource requirements:

1. Pharmaceutical management only: Cases where only medications (analgesics, antibiotics) were prescribed without any dental procedure
2. Temporary restoration: Placement of temporary filling materials, including intermediate restorative materials
3. Permanent restoration: Definitive restorative procedures using amalgam, composite, or glass ionomer materials
4. Pulp therapy: Including pulpotomy, pulpectomy, and pulp extirpation procedures
5. Extraction: Removal of primary or permanent teeth
6. Drainage: Incision and drainage procedures for abscesses

For analytical purposes, these treatments were further grouped into "conservative" (categories 1–3) and "invasive" (categories 4–6) approaches to examine shifts in treatment strategies across the study periods.

Detailed breakdowns of emergency visit characteristics by age group and time period are provided in supplementary materials to ensure complete transparency of data presentation.

### Statistical analysis

Statistical analysis was performed using R software (R Core Team, 2024, Vienna, Austria). Basic and descriptive analyses utilized the stats, dplyr, and tidyr packages. Age-related differences were analysed using ANOVA with Tukey HSD post-hoc tests. Chi-square tests were used to compare the distribution of reasons for emergency visits and types of treatments across age groups and time periods.

To test for interactions between age groups and treatment periods, we included interaction terms in the logistic regression models and conducted ANCOVA analysis to examine whether the effect of age on treatment selection differed across the three time periods. This approach allowed us to determine if certain age groups, particularly the youngest children (0–3 years), were disproportionately affected by changes in treatment approaches during the lockdown period.

For categorical variables, chi-square tests of independence were used to examine differences in proportions between time periods and age groups. The chi-square test was applied to assess whether the distribution of age groups differed significantly across the three study periods.

In all statistical tests, a *p*-value less than 0.05 was considered statistically significant. For multiple comparisons, Bonferroni correction was applied to prevent type I errors.

## Results

### Study population characteristics

A total of 6,024 emergency dental visits for children under 12 years were analysed across the three study periods. During the pre-COVID period (March–May 2019), 2,331 visits were recorded. This number decreased significantly to 1,393 visits during the lockdown period (March–May 2020), representing a 40.2% reduction. In the post-lockdown period (March–May 2021), the number of visits returned to pre-pandemic levels with 2,300 visits.

Analysis of patient age revealed significant differences between periods. One-way ANOVA showed a statistically significant difference in mean age across the three periods (*F* = 42.3, *p* < 0.001). Post-hoc Tukey HSD analysis demonstrated that the mean age during the lockdown period (6.2 years, SD = 2.8) was significantly lower than both the pre-COVID period (7.1 years, SD = 2.9, *p* < 0.001) and the post-lockdown period (6.8 years, SD = 2.7, *p* < 0.001). Further analysis revealed that the mean age in the post-lockdown period remained significantly lower than the pre-COVID period (*p* = 0.03). Sex distribution remained consistent across all periods, with approximately 53% male and 47% female patients (*χ*^2^ = 1.74, df = 2, *p* = 0.42). The demographic characteristics of the study population are presented in Table 1.

**Table 1:** Demographic characteristics of the study population by period

Characteristic	Pre-COVID period	Lockdown period	Post-lockdown period	*p*-value
Number of visits	2331	1393	2300	–
Mean age (SD), years	7.1 (2.9)	6.2 (2.8)	6.8 (2.7)	< 0.001*
Sex, *n* (%)				0.42†
– Male	1.235 (53.0%)	738 (53.0%)	1.219 (53.0%)	
– Female	1.096 (47.0%)	655 (47.0%)	1.081 (47.0%)	

*One-way ANOVA; †Chi-square test for independence

### Age-specific patterns in emergency visits

When analysing emergency visit patterns by age group, the 0–3 years group showed distinct characteristics during the lockdown period. As shown in Table 2, there was a disproportionate representation of very young children (0–3 years) among emergency visits during the lockdown period compared to other periods. While the overall number of emergency visits decreased by 40.2% during lockdown, a clear age gradient was observed in the reduction rates. The 0–3 age group showed the smallest relative decrease (35.7% reduction), while the 10–12 age group experienced the most dramatic decrease with a 52.0% reduction compared to the pre-COVID period (150 visits vs. 396 visits). Chi-square analysis revealed statistically significant differences in the age group distribution between the three study periods (*χ*^2^ = 35.912, df = 6, *p* < 0.001). The 10–12 years age group experienced the most dramatic reduction in emergency visits during lockdown (62.1% decrease), which was significantly greater than all other age groups. In contrast, the younger age groups (0–3, 4–6, and 7–9 years) showed remarkably similar reductions (35.8%, 35.3%, and 36.2%, respectively), all of which were substantially lower than the oldest age group. This pattern demonstrates a clear age gradient in service utilization during the healthcare crisis, with older children showing greater reduction in emergency dental visits compared to younger children (*p* < 0.001).

**Table 2:** Distribution of emergency dental visits by age group and period

Age group (years)	Pre-COVID period	Lockdown period	Post-lockdown period	% change during lockdown*	*p*-value†
0–3	466 (20.0%)	299 (21.5%)	416 (18.1%)	− 35.8%	< 0.001
4–6	781 (33.5%)	505 (36.3%)	772 (33.6%)	− 35.3%	< 0.001
7–9	688 (29.5%)	439 (31.5%)	722 (31.4%)	− 36.2%	< 0.001
10–12	396 (17.0%)	150 (10.8%)	390 (17.0%)	− 62.1%	< 0.001
Total	2331 (100%)	1393 (100%)	2300 (100%)	− 40.2%	< 0.001‡

*Percent change calculated relative to pre-COVID period

†Chi-square test comparing proportions between periods

‡Chi-square test comparing age group distribution between periods (*χ*^2^ = 35.912, df = 6, *p* < 0.001)

The mean daily number of emergency visits also showed significant variation between periods and age groups. One-way ANOVA confirmed a significant difference in daily visits across periods (*F* = 98.4, df = 2, *p* < 0.001). During the lockdown period, the mean daily number of emergency visits was 34.1 (SD = 12.4), significantly lower than the pre-COVID period (57.1, SD = 8.7, *p* < 0.001) and post-lockdown period (56.1, SD = 9.1, *p* < 0.001).

Complete data for reasons for emergency visits across all age groups and time periods are presented in Supplementary Table S1. This comprehensive analysis confirms that the 0–3 years age group demonstrated the most consistent vulnerability patterns across all study periods, with the smallest reduction in emergency visits during lockdown (-35.8%) and stable proportions of severe conditions (dental pain and swelling/abscess) regardless of external circumstances.

### Reasons for emergency visits by age group

Analysis of reasons for emergency visits revealed significant age-specific patterns, with the 0–3 years group showing distinct characteristics during the lockdown period. As shown in Table 3, children in this youngest age group had the highest proportion of visits due to dental pain (51.2%) and swelling/abscess (24.8%) during the lockdown period, both representing significant increases compared to the pre-COVID period (42.1% and 19.5%, respectively, *p* < 0.001 and *p* = 0.03).

**Table 3:** Reasons for emergency dental visits by age group during the lockdown period

Reason for Visit	0–3 years	4–6 years	7–9 years	10–12 years	Total	*p*-value*
Dental pain	128 (51.2%)	208 (44.3%)	168 (38.2%)	92 (36.8%)	596	< 0.001
Soft tissue pain	34 (13.6%)	74 (15.8%)	75 (17.1%)	38 (15.2%)	221	0.74
Dental trauma	13 (5.2%)	41 (8.7%)	45 (10.2%)	18 (7.2%)	117	< 0.001
Swelling and abscess	62 (24.8%)	90 (19.1%)	76 (17.3%)	39 (15.6%)	267	0.02
Repair of existing treatment	13 (5.2%)	57 (12.1%)	75 (17.2%)	63 (25.2%)	192	< 0.001
Total	299 (100%)	505 (100%)	439 (100%)	150 (100%)	1393	–

*Chi-square test comparing distribution across age groups

When comparing the lockdown period to the pre-COVID period, multivariate analysis revealed that the odds of presenting with dental pain were significantly higher during lockdown (OR = 1.64, 95% CI: 1.42–1.89, *p* < 0.001), as were the odds of presenting with swelling or abscess (OR = 1.37, 95% CI: 1.18–1.62, *p* < 0.001). Conversely, the odds of presenting with dental trauma were significantly lower (OR = 0.41, 95% CI: 0.33–0.51, *p* < 0.001).

Table 4 presents a comparative analysis of reasons for emergency visits in the 0–3 years age group across the three time periods, highlighting the significant changes observed during the lockdown period. When comparing the pre-COVID period directly with the post-lockdown period for the 0–3 age group, the differences were less pronounced than during the lockdown. Dental pain presentations remained slightly elevated in the post-lockdown period (43.8%) compared to pre-COVID (42.1%), though this difference was not statistically significant (*p* = 0.76). Similarly, the difference in trauma-related visits between pre-COVID and post-lockdown periods (12.4% vs. 11.8%) was not statistically significant (*p* = 0.76), nor was the reduction in repair of existing treatment (11.8% vs. 9.9%, *p* = 0.35).

**Table 4:** Comparison of reasons for emergency visits in 0–3 years age group across periods

Reason for Visit	Pre-COVID Period	Lockdown period	Post-lockdown period	Total	*p*-value*
Dental pain	196 (42.1%)	128 (51.2%)	182 (43.8%)	506	< 0.001
Soft tissue pain	66 (14.2%)	34 (13.6%)	60 (14.5%)	160	0.86
Dental trauma	58 (12.4%)	13 (5.2%)	49 (11.8%)	120	< 0.001
Swelling and abscess	91 (19.5%)	62 (24.8%)	84 (20.2%)	137	0.03
Repair of existing treatment	55 (11.8%)	13 (5.2%)	41 (9.7%)	158	0.02
Total	466 (100%)	299 (100%)	416 (100%)	1181	–

*Chi-square test comparing distribution across periods

### Treatment types and invasive interventions by age group

Analysis of treatment modalities revealed that children in the 0–3 years age group received a significantly higher proportion of invasive treatments during the lockdown period compared to other age groups. As shown in Table 5, the proportion of extractions in the 0–3 years group during lockdown (24.8%) was significantly higher than in other age groups and higher than in the pre-COVID period (18.4%, *p* < 0.001). Notably, permanent restorations showed a distinctive pattern across age groups, with percentages increasing steadily from the youngest children (7.7% in 0–3 years) to the oldest group (16.7% in 10–12 years). This was the only treatment category that demonstrated a consistent upward trend with increasing age (*p* < 0.001), contrasting with other treatments that showed either decreasing trends or non-linear patterns across age groups.

**Table 5:** Types of treatments provided by age group during the lockdown period

Treatment Type	0–3 years	4–6 years	7–9 years	10–12 years	Total	*p*-value*
Pharmacological treatment only	67 (22.4%)	114 (22.5%)	93 (21.2%)	38 (25.3%)	312	0.71
Temporary restoration	61 (20.4%)	109 (21.6%)	98 (22.3%)	30 (20.0%)	298	0.88
Permanent restoration	23 (7.7%)	62 (12.3%)	61 (13.9%)	25 (16.7%)	171	< 0.001
Pulp extirpation	59 (19.7%)	79 (15.6%)	58 (13.2%)	21 (14.0%)	217	0.02
Extraction	74 (24.8%)	124 (24.6%)	104 (23.7%)	43 (28.7%)	345	0.64
Drainage	15 (5.0%)	17 (3.4%)	25 (5.7%)	3 (2.0%)	50	0.11
Total	299 (100%)	505 (100%)	439 (100%)	150 (100%)	1393	–

*Chi-square test comparing distribution across age groups

Multivariate logistic regression analysis was performed to evaluate factors influencing the likelihood of receiving invasive treatment (Table 6). The model included time period, age group, sex, and presenting complaint as independent variables, with pre-COVID period, 10–12 years age group, female sex, and issues with existing treatment serving as reference categories, respectively. Interaction terms between time period and age group were also included to test for age-specific effects of the lockdown. The model showed good fit (Hosmer–Lemeshow test: *χ*^2^ = 11.2, df = 8, *p* = 0.19) and reasonable explanatory power (Nagelkerke *R*^2^ = 0.24).

**Table 6:** Multivariate logistic regression analysis for factors influencing invasive treatment during lockdown

Variable	Adjusted odds ratio	95% CI	*p*-value
Time period			
Pre-COVID (reference)	1.00	–	-
Lockdown	1.82	1.54–2.15	< 0.001
Post-lockdown	1.02	0.86–1.18	0.84
Age group			
0–3 years	1.38	1.12–1.68	0.008
4–6 years	1.15	0.94–1.41	0.17
7–9 years	1.09	0.88–1.34	0.42
10–12 years (reference)	1.00	–	–
Sex			
Female (reference)	1.00	–	2013
Male	1.07	0.94–1.22	0.31
Presenting complaint			
Dental pain	1.64	1.42–1.89	< 0.001
Soft tissue pain	0.96	0.82–1.21	0.74
Trauma	0.41	0.33–0.51	< 0.001
Swelling/abscess	1.37	1.18–1.62	< 0.001
Issues with existing treatment (reference)	1.00	–	–
Interaction terms			
Lockdown × 0–3 years	1.38	1.12–1.68	0.008
Model Nagelkerke *R*^2^	0.24		

Table 7 presents a detailed comparison of treatment types in the 0–3 years age group across the three study periods. This age group showed particularly distinctive patterns in treatment distribution during the lockdown. A particularly noteworthy finding was the continued decline in pharmaceutical-only management in the post-lockdown period for the 0–3 age group, decreasing further from 22.4% during lockdown to 15.6% post-lockdown. This represents a 63.8% overall reduction from the pre-COVID baseline (43.1%). Concurrently, invasive treatments increased significantly, with pulp therapy rising from 12.2% pre-COVID to 19.7% during lockdown (*p* = 0.003) and extractions increasing from 18.4 to 24.8% (*p* = 0.03). The provision of permanent restorations showed the most substantial decrease, from 17.0% pre-COVID to only 7.7% during lockdown (*p* < 0.001), representing a 54.7% reduction in definitive restorative treatment for this age group.

**Table 7:** Comparison of treatment types in 0–3 years age group across periods

Treatment Type	Pre-COVID period	Lockdown period	Post-lockdown period	*p*-value*
Pharmacological treatment only	201 (43.1%)	67 (22.4%)	65 (15.6%)	< 0.001
Temporary restoration	135 (29.0%)	61 (20.4%)	125 (30.0%)	0.02
Permanent restoration	79 (17.0%)	23 (7.7%)	71 (17.1%)	< 0.001
Pulp extirpation	57 (12.2%)	59 (19.7%)	48 (11.5%)	0.003
Extraction	86 (18.4%)	74 (24.8%)	82 (19.7%)	0.03
Drainage	38 (8.2%)	15 (5.0%)	25 (6.0%)	0.19

*Chi-square test comparing distribution across periods

**Fig. 1:**
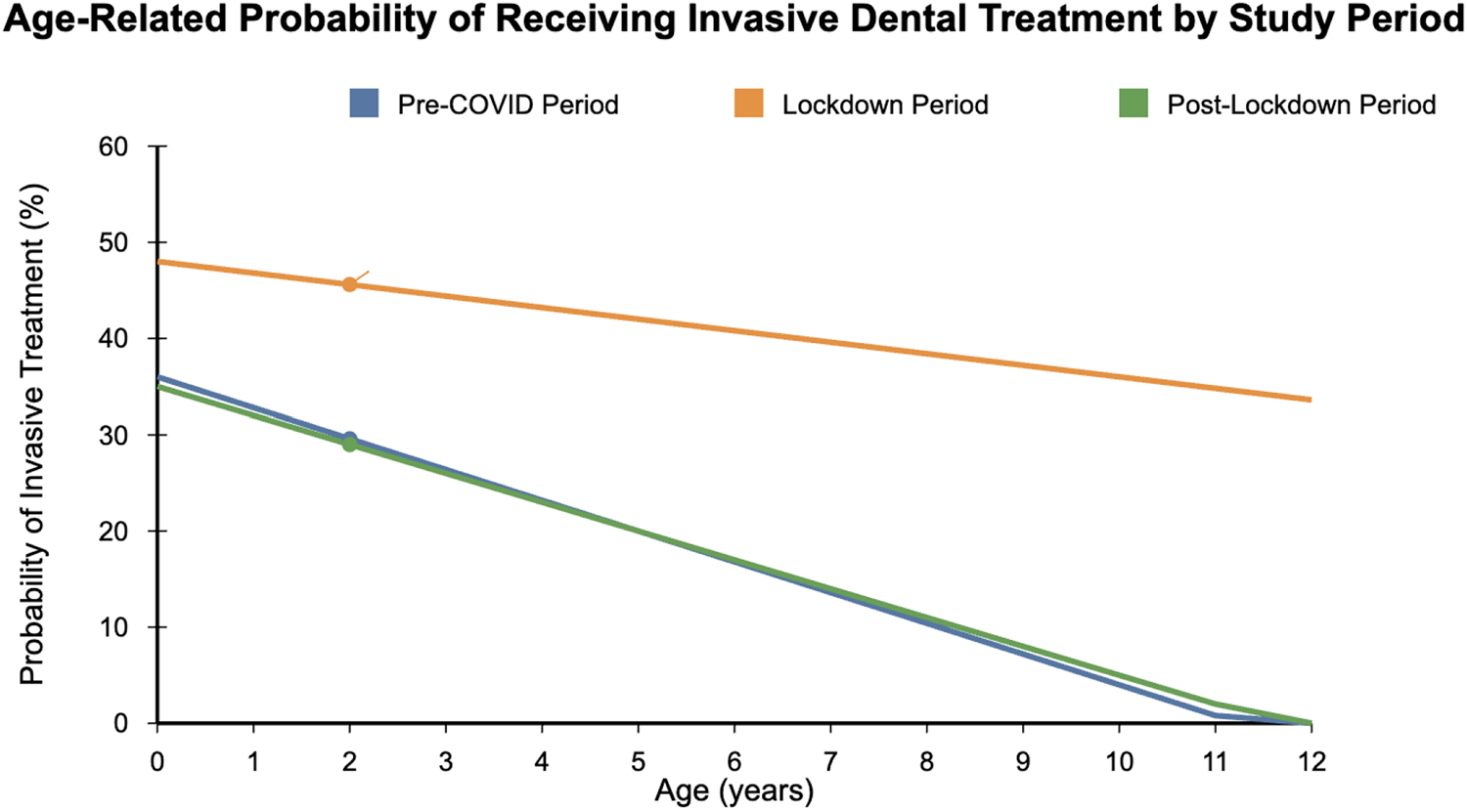
Age-related probability of receiving invasive dental treatment (extraction or pulp extirpation) by study period. The graph illustrates the relationship between patient age and likelihood of invasive treatment, demonstrating a less pronounced age-related gradient during the lockdown period (*β* = − 0.06) compared to pre-COVID (*β* = − 0.14) and post-lockdown periods (*β* = − 0.13)

ANCOVA analysis examining the effect of age on the likelihood of receiving invasive treatment, with period as a factor, revealed a significant main effect of age (*F* = 45.7, df = 1, *p* < 0.001) and a significant interaction between age and period (*F* = 12.8, df = 2, *p* < 0.001). As illustrated in Fig. 1, the negative relationship between age and likelihood of invasive treatment was stronger in the pre-COVID period (*β* = − 0.14, *p* < 0.001) and post-lockdown period (*β* = − 0.13, *p* < 0.001) than during the lockdown period (*β* = − 0.06, *p* = 0.08). This indicates that while younger children generally have a higher likelihood of requiring invasive treatments, this age-related gradient was less pronounced during the lockdown period due to the overall increase in invasive treatments across all age groups, with particularly high rates among the youngest children.

## Discussion

This study identified significant age-specific vulnerability patterns in paediatric dental emergencies during the COVID-19 lockdown, with very young children (0–3 years) demonstrating unique emergency needs and treatment outcomes. Despite an overall 40.2% reduction in paediatric dental emergencies during lockdown, the decrease was less pronounced in the youngest age group (35.7%) compared to older children, especially the 10–12 age group (52.0%). This pattern aligns with findings from similar studies worldwide (Alzahrani et al. 2021; Eggmann et al. 2021; Fux-Noy et al. 2021).

The significant drop in mean age of patients seeking emergency dental care during lockdown (from 7.1 to 6.2 years) confirms younger children's particular vulnerability during healthcare disruptions. This finding is consistent with research from Switzerland (Eggmann et al. 2021), Saudi Arabia (Alzahrani et al. 2021), and Argentina (Rodriguez et al. 2022), suggesting a consistent pattern across different healthcare systems.

The observed age gradient in emergency visit reduction, with older children (10–12 years) showing the most substantial decrease (52.0%) while the youngest age group (0–3 years) showed the smallest reduction (35.7%), suggests age-specific differences in care-seeking behavior during the crisis. This pattern may reflect several factors: parents may perceive dental problems in younger children as more urgent and requiring immediate attention regardless of external circumstances (Samuel et al. 2021); older children may be better able to tolerate or communicate about mild to moderate dental discomfort, allowing parents to defer care during lockdown (Goswami et al. 2021); and older children typically have fewer developmental dental issues requiring emergency intervention (Masri et al. 2021). Additionally, the anatomical structure of primary teeth in younger children, with thinner enamel and larger pulp chambers, allows for more rapid progression of dental pathology (Luzzi et al. 2021), potentially creating more urgent situations that families felt unable to postpone despite pandemic restrictions. These findings reinforce the particular vulnerability of very young children in dental emergency contexts during healthcare crises (Sudri et al. 2024) and highlight the need for age-tailored approaches in emergency dental service planning (Whyte et al. 2023).

A concerning finding was the increased proportion of emergency visits due to dental pain (51.2%) and swelling/abscess (24.8%) among 0–3-year-olds during lockdown compared to pre-COVID levels (42.1% and 19.5%, respectively). This pattern mirrors previous research, which reported a higher prevalence of pulpal pathology emergencies among younger children during lockdown (Goswami et al. 2021; Kumar et al. 2021). Several factors likely explain this increased prevalence of pulpal pathology. First, dental care for very young children was often considered non-urgent pre-pandemic, with many practitioners opting for watchful waiting of early carious lesions (Luzzi et al. 2021). During lockdown, this approach resulted in rapid progression to symptomatic disease, particularly in primary teeth with their larger pulp chambers and thinner enamel. Second, pandemic-related changes in diet and oral hygiene routines may have accelerated caries development, with families reporting increased consumption of cariogenic snacks and disrupted oral hygiene supervision during home confinement (Goswami et al. 2021). Finally, pandemic-related stress and altered sleep patterns in young children may have exacerbated bruxism and other parafunctional habits, potentially accelerating pulpal involvement in teeth with pre-existing conditions (Samuel et al. 2019).

The dramatic reduction in trauma-related visits across all age groups, but particularly in the 0–3 group (from 12.4 to 5.2%), was expected due to reduced physical activity during lockdown and corroborates findings from multiple studies (Olszewska et al. 2021; Üstün et al. 2021; Yang and Yoon 2023).

The most concerning finding was the significant increase in invasive treatments in the 0–3 age group during lockdown. The proportion of extractions rose from 18.4% pre-COVID to 24.8% during lockdown (*p* = 0.03), while pulp extirpations increased from 12.2% to 19.7% (*p* = 0.003). The interaction effect identified in our multivariate model (OR = 1.38, 95% CI: 1.12–1.68, *p* = 0.008) suggests very young children had a disproportionately greater likelihood of requiring invasive treatments during lockdown. Similar patterns have been observed in other studies, including increased rates of pulp therapy and extractions among young children in Germany (Masri et al. 2021) and increased abscess drainages in Poland (Olszewska et al. 2021).

The observed steady increase in permanent restorations with age (from 7.7% in the 0–3 age group to 16.7% in the 10–12 age group) reflects important developmental and clinical considerations in paediatric dental practice. This pattern likely stems from multiple factors: the greater structural integrity of teeth in older children making them more suitable candidates for definitive restorations; the increased cooperative capacity of older children allowing for more technique-sensitive procedures; and the longer remaining lifespan of permanent teeth in older children justifying more definitive interventions (Goswami et al. 2021; Luzzi et al. 2021). Conversely, the lower rates of permanent restorations in the youngest age group highlight another dimension of their vulnerability during emergencies—not only did they experience more severe conditions requiring intervention, but they were also less likely to receive definitive restorative treatments that might prevent future emergencies, potentially creating a cycle of repeated emergency visits.

The age-related gradient in treatment invasiveness provides further insight into this vulnerability pattern. The weaker negative relationship between age and likelihood of invasive treatment during lockdown (*β* = − 0.06) compared to pre-COVID (*β* = − 0.14) and post-lockdown periods (*β* = − 0.13) demonstrates how the healthcare crisis intensified age-based disparities in treatment needs.

The continued decline in pharmaceutical-only management for the 0–3 age group even after lockdown restrictions were lifted (from 43.1% pre-COVID to 15.6% post-lockdown) may represent a lasting effect of the pandemic on paediatric dental practice. While this pattern could suggest a shift in treatment philosophy, alternative explanations should be considered, including changes in case severity, evolving clinical guidelines, or methodological factors. Longer-term follow-up studies would be needed to confirm whether this represents a permanent change in clinical practice. Several factors might explain this lasting change: dental practitioners may have observed better outcomes with more definitive interventions during the lockdown period; the experience of uncertainty about future care access may have permanently altered risk assessment calculations; or the pandemic may have accelerated an existing trend toward more definitive management of dental emergencies in very young children (Luzzi et al. 2021; Masri et al. 2021). Regardless of the underlying cause, this finding indicates that certain aspects of crisis-induced changes in clinical decision-making may have longer-term impacts on treatment patterns, outlasting the immediate conditions that prompted them.

Several factors likely contribute to very young children's unique vulnerability pattern. The anatomical structure of primary teeth, characterized by thinner enamel and wider root canals, allows more rapid progression of dental caries (Luzzi et al. 2021). Limited ability to communicate pain severity often leads to delayed recognition of dental problems until they become severe (Goswami et al. 2021; Samuel et al. 2019). Additionally, different immune responses in very young children may lead to faster progression of dental infections (Sudri et al. 2024). During lockdown, these factors were likely exacerbated by heightened parental concerns about virus exposure (Cagetti et al. 2021; Remmani et al. 2023).

Our findings have important implications for both crisis response and routine dental care. The unique vulnerability patterns observed in very young children (0–3 years) during the pandemic highlight characteristics that remain relevant in normal circumstances: anatomical features predisposing primary teeth to rapid caries progression (Luzzi et al. 2021), limited communication abilities hampering timely pain reporting (Samuel et al. 2019), and developmental challenges in treatment cooperation (Goswami et al. 2021). While many dental conditions could be deferred during healthcare crises, the disproportionate impact on the youngest age group indicates this population requires special consideration in emergency planning (Dave et al. 2020; Whyte et al. 2023). These findings suggest dental care systems should restructure preventive protocols to place greater emphasis on early childhood, with more frequent recall intervals and prioritized access to specialist services (Masri et al. 2021). The consistently high proportion of pulpal pain and abscess presentations in very young children points to potential gaps in preventive dental care for this age group (Choi et al. 2024).

Implementing age-specific pathways that acknowledge the distinctive needs of very young children could reduce emergency presentations and invasive treatments even during non-crisis periods, improving both oral health outcomes and resource utilization.

Several limitations should be considered when interpreting these findings. First, the retrospective design limits our ability to establish causality between lockdown measures and observed changes in emergency presentations. Second, data from a single healthcare provider, though serving approximately 25% of Israel's population, may limit generalizability. Third, we lacked detailed information on socioeconomic factors, parental education, and home oral hygiene practices, which could influence pre-existing conditions and care-seeking behaviours (Choi et al. 2024; Siboro et al. 2023). Fourth, our study focused exclusively on emergency visits and did not capture potential long-term consequences of delayed routine care. Finally, we lack information about emergency dental visits to private clinics or those managed through teledentistry during lockdown (Hung et al. 2022). Finally, the study also has limitations in its ability to assess the impact of socioeconomic, cultural, and social factors on emergency visit patterns. While the data include basic geographic and demographic information, detailed information about socioeconomic status, parental education, and social determinants of health that could influence care-seeking behavior during the pandemic is lacking. Additionally, the study does not capture information about specific barriers that prevented families from seeking care, such as transportation difficulties, financial constraints, or health-related concerns about COVID-19 exposure.

Future research should focus on several key areas. Longitudinal studies following children who received emergency dental treatment during lockdown would provide valuable insights into the long-term consequences of delayed care and invasive treatments in the youngest age group. Investigation of age-specific risk factors for severe dental emergencies, combining clinical data with detailed demographic and behavioural information, could help identify modifiable risk factors and develop targeted preventive strategies. More granular analysis of age-specific vulnerability patterns would be particularly valuable. While our study categorized children into broad age groups, even within the 0–3 years category, there likely exist meaningful developmental differences affecting both disease progression and care-seeking behaviour. For instance, children aged 2–3 years, who have more developed communication abilities, may better articulate dental pain compared to infants and very young toddlers, potentially leading to earlier intervention. Similarly, the presence of a complete primary dentition versus a partially erupted one may influence both caries patterns and treatment approaches. Future studies with larger sample sizes should consider more refined age categorizations (e.g., 0–12, 13–24, 25–36 months) to better characterize vulnerability periods and inform age-specific preventive protocols. Also, they should incorporate comprehensive socioeconomic and behavioral data to better understand the intersection between social determinants of health and age-specific vulnerabilities in pediatric dental emergencies. Research examining the role of parental education, household income, insurance coverage, and cultural factors in care-seeking behavior during healthcare crises would provide valuable insights for developing targeted interventions.

The effectiveness of alternative care delivery models, such as teledentistry triage, should be evaluated specifically for different paediatric age groups. While such approaches showed promise during the pandemic, their effectiveness for managing dental emergencies in very young children requires further investigation. Finally, comparative studies across different healthcare systems would enhance understanding of how system-level factors influence age-specific vulnerability patterns in paediatric dental emergencies.

## Conclusion

This study demonstrates that very young children (0–3 years) exhibited unique vulnerability patterns during the COVID-19 lockdown, with a less pronounced reduction in emergency visits compared to older children. The significantly higher rates of dental pain (51.2%) and abscess (24.8%) presentations, coupled with increased need for invasive treatments (44.5% requiring extraction or pulp therapy versus 30.6% pre-COVID), indicate that this age group experienced more severe consequences from delayed dental care. These findings highlight the need for age-specific emergency protocols that recognize the unique characteristics of very young children and suggest that maintaining accessible emergency dental services for this population should be prioritized during healthcare disruptions. Future emergency preparedness planning should include dedicated pathways for children under 4 years to mitigate adverse outcomes during similar healthcare crises.

## Supplementary Information

Below is the link to the electronic supplementary material.

Supplementary file1 (DOCX 17 kb)

## Data Availability

The data that support the findings of this study are available from Maccabi Healthcare Services, but restrictions apply to the availability of these data, which were used under license for the current study, and so are not publicly available. Data are, however, available from the authors upon reasonable request and with permission of Maccabi Healthcare Services.
