# Supplementary material for: Age-specific vulnerabilities in paediatric dental emergencies before, during, and after COVID-19 lockdown: a retrospective comparative analysis with emphasis on early childhood (0–3 years)
Source: Eur Arch Paediatr Dent. 2025 Jul 29;27(2):463–73. doi: 10.1007/s40368-025-01088-5 (PMC13083354; doi:10.1007/s40368-025-01088-5)
Supplement: Supplementary file 1 — Supplementary file1 (DOCX 17 kb) [file 40368_2025_1088_MOESM1_ESM.docx]

**Supplementary Table S1: Reasons for Emergency Dental Visits by Age Group and Period (Complete Analysis)**

**Part A: Pre-COVID Period (March-May 2019)**

| **Reason for Visit** | **0-3 years** | **4-6 years** | **7-9 years** | **10-12 years** | **Total** |
| --- | --- | --- | --- | --- | --- |
| Dental pain | 196 (42.1%) | 243 (31.1%) | 182 (26.5%) | 101 (25.5%) | 722 |
| Soft tissue pain | 66 (14.2%) | 133 (17.0%) | 121 (17.6%) | 64 (16.2%) | 384 |
| Dental trauma | 58 (12.4%) | 139 (17.8%) | 157 (22.8%) | 82 (20.7%) | 436 |
| Swelling and abscess | 91 (19.5%) | 119 (15.2%) | 85 (12.4%) | 50 (12.6%) | 345 |
| Repair of existing | 55 (11.8%) | 147 (18.8%) | 143 (20.8%) | 99 (25.0%) | 444 |
| **Total** | **466 (100%)** | **781 (100%)** | **688 (100%)** | **396 (100%)** | **2,331** |

**Part B: Lockdown Period (March-May 2020)**

| **Reason for Visit** | **0-3 years** | **4-6 years** | **7-9 years** | **10-12 years** | **Total** |
| --- | --- | --- | --- | --- | --- |
| Dental pain | 128 (42.8%) | 224 (44.4%) | 168 (38.3%) | 76 (50.7%) | 596 |
| Soft tissue pain | 34 (11.4%) | 80 (15.8%) | 75 (17.1%) | 32 (21.3%) | 221 |
| Dental trauma | 13 (4.3%) | 44 (8.7%) | 45 (10.3%) | 15 (10.0%) | 117 |
| Swelling and abscess | 62 (20.7%) | 96 (19.0%) | 76 (17.3%) | 33 (22.0%) | 267 |
| Repair of existing | 62 (20.7%) | 61 (12.1%) | 75 (17.1%) | 24 (16.0%) | 222 |
| **Total** | **299 (100%)** | **505 (100%)** | **439 (100%)** | **150 (100%)** | **1,393** |

**Part C: Post-Lockdown Period (March-May 2021)**

| **Reason for Visit** | **0-3 years** | **4-6 years** | **7-9 years** | **10-12 years** | **Total** |
| --- | --- | --- | --- | --- | --- |
| Dental pain | 182 (43.8%) | 259 (33.5%) | 208 (28.8%) | 122 (31.3%) | 771 |
| Soft tissue pain | 60 (14.4%) | 130 (16.8%) | 128 (17.7%) | 68 (17.4%) | 386 |
| Dental trauma | 49 (11.8%) | 127 (16.5%) | 150 (20.8%) | 70 (17.9%) | 396 |
| Swelling and abscess | 84 (20.2%) | 109 (14.1%) | 86 (11.9%) | 45 (11.5%) | 324 |
| Repair of existing | 41 (9.9%) | 147 (19.0%) | 150 (20.8%) | 85 (21.8%) | 423 |
| **Total** | **416 (100%)** | **772 (100%)** | **722 (100%)** | **390 (100%)** | **2,300** |
